# Correction: Geographical variation in the diatom communities associated with loggerhead sea turtles (*Caretta caretta*)

**DOI:** 10.1371/journal.pone.0247350

**Published:** 2021-02-17

**Authors:** The PLOS ONE Staff

In Table 1, the data on seven samples are missing. The publisher apologizes for the error. Please see the correct Table 1 here.

**Table 1:** List of samples and information on loggerhead sea turtles.

Sample code	Body part	ID Tag / Turtle name	Sampling Date	Sex	Weight (kg)	SCL (cm)	CCL (cm)	SCW (cm)	CCW (cm)
**Sampling location: Amvrakikos Gulf (Greece) 39° 1’ 29" N—39° 1’ 44" N; 21° 3’ 36" E—21° 4’ 19" E**									
GRE-01	carapace	Y6343- Y6344	8/1/2018			75.4	78.6	55.0	68.5
GRE-02	skin	Y6343- Y6344							
GRE-03	carapace	Y6366- Y6367	8/1/2018			47.4	51.0	36.3	46.8
GRE-04	skin	Y6366- Y6367							
GRE-05	carapace	Y6368- Y6369	8/1/2018			66.1	69.6	51.5	63.8
GRE-06	skin	Y6368- Y6369							
GRE-07	carapace	Y6370- Y6371	8/1/2018			54.5	58.5	43.7	55.2
GRE-08	skin	Y6370- Y6371							
GRE-09	carapace	M9123- M9124	8/1/2018			50.4	53.2	37.5	47.5
GRE-10	skin	M9123- M9124							
**Sampling location: Florida Bay (USA) 24° 55’18” N; 80° 48’ 28” W**									
FLO-U8	carapace	HA5053—HA5054	6/24/2015	F	58.5	74.1			
FLO-U9	carapace	HB5559—HB5560	6/24/2015		48.0	66.9			
FLO-U10	carapace	HB5668—X7596	6/24/2015	M	89.0	87.3			
FLO-U11	carapace	W1924—W2176	6/24/2015	F	75.7	79.5			
FLO-U12	carapace	HB5581—HB5582	6/25/2015		71.2	78.5			
**Sampling location: Kosi Bay (South Africa) 26° 59’ 38.9" S; 32° 51’ 59.8" E**									
SA-33	carapace	ZA0447D - ZA0427D	1/16/2018	F			86.4		83.6
SA-37	carapace	ZA0829D - ZA0828D	1/11/2018	F		80.2	85.7	62.0	78.8
SA-45	carapace	ZA0924D - ZA0186D	1/11/2018	F		73.9	80.2	58.2	74.8
SA-47	carapace	ZA0833D - ZA1923D	1/13/2018	F			91.4		81.8
SA-55	carapace	ZA1406D - ZA1407D	1/18/2018	F		84.0	92.7	64.0	88.5
**Sampling location: Adriatic Sea (Croatia) 44° 50’ 07” N; 13° 49’ 58” E**									
CRO-13	carapace	Zanja-Mara	5/30/2016		11.0	44.0	43.0		41.0
CRO-19	carapace	Lunga	8/30/2016		28.8	69.8	68.0		63.2
CRO-21	carapace	Palma	9/30/2016	F	45.0	70.2	68.5		71.5
CRO-24	carapace	Shiggy-Lola	12/5/2016		0.9	24.1	24.5		23.0
CRO-42	carapace	Tylago	2/24/2017		28.2	64.0	63.0		58.0

CCL = curved carapace length, CCW = curved carapace width, SCL = straight carapace length, SCW = straight carapace width
