# Supplementary material for: Geographical variation in the diatom communities associated with loggerhead sea turtles (Caretta caretta)
Source: PLoS One. 2020 Jul 29;15(7):e0236513. doi: 10.1371/journal.pone.0236513 (PMC7390603; doi:10.1371/journal.pone.0236513)
Supplement: S1 Table — (PDF) [file pone.0236513.s001.pdf]

S1 Table. **List of taxonomic publications used for identification of diatom taxa on loggerhead sea turtles.**

Monographs

Álvarez–Blanco I, Blanco S (2014) Benthic diatoms from Mediterranean coasts. Bibl. Diatomol. 60: 1–409

Foged N (1975) Some Littoral Diatoms from the Coast of Tanzania. Bibl Phycol 16: 1–127

Hein, MK, Winsborough, BM, Sullivan, MJ (2008) Bacillariophyta (diatoms) of the Bahamas Iconographia Diatomologica 19: 1–303

Loir M, Novarina G (2013) Marine *Mastogloia* Thwaites ex W.Sm. and *Stigmaphora* Wallich species from the French Lesser Antilles. Diatom Monogr 16: 1–133

Witkowski A, Lange–Bertalot H, Metzeltin D (2000) Diatom Flore of Marine Coasts I. Icon Diatomol 7: 1–925

Articles

Ashworth MP, Ruck EC, Lobban CS, Romanovicz DK, Theriot EC (2012) A revision of the genus *Cyclophora* and description of *Astrosyne* gen. nov. (Bacillariophyta), two genera with the pyrenoids contained within pseudosepta. Phycologia 51: 684–699

Belando MD, Jimenez JF, Marín A, Aboal M (2018) Morphology and molecular phylogeny of *Hyalosynedra lanceolata* sp. nov. and an extended description of *Hyalosynedra* (Bacillariophyta). Eur J of Phycol 53: 208–218

Belando MD, Marín A, Aboal M (2012) *Licmophora* species from a Mediterranean hypersaline coastal lagoon (Mar Menor, Murcia, SE Spain) Nova Hedwigia 141: 275–288

Cremer H, Sangiorgi F, Wagner–Cremer F, McGee V, Lotter AF, Visscher H (2007) Diatoms (Bacillariophyceae) and Dinoflagellate Cysts (Dinophyceae) from Rookery Bay, Florida, USA. Caribb J Sci 43: 23–58

Danielidis D, Mann D (2003) New species and new combinations in the genus *Seminavis* (Bacillariophyta). Diatom Res 18: 21–39

Frankovich T, Wachnicka A (2015) Epiphytic Diatoms along Phosphorus and Salinity Gradients in Florida Bay (Florida, USA), an Illustrated Guide and Annotated Checklist In Microbiology of the Everglades Ecosystem. In: Entry JA, Gottlieb AD, Jayachandran K, Ogram A (eds) Microbiology of the Everglades Ecosystem, CRC Press, Boca Raton, pp. 239–286

Frankovich TA, Ashworth MP, Sullivan MJ, Veselá J, Stacy NI (2016) *Medlinella amphoroidea* gen. et sp. nov. (Bacillariophyta) from the neck skin of Loggerhead sea turtles (*Caretta caretta*). Phytotaxa 272: 101–114

Frankovich TA, Sullivan MJ, Stacy MI (2015) Three new species of *Tursiocola* (Bacillariophyta) from the skin of the West Indian manatee (*Trichechus manatus*). Phytotaxa 204: 33–48

Gaiser EE, Wachnicka AH, Taylor C, Travieso R (2006) Diatom–based water quality performance metrics for Biscayne Bay. Florida International University 1–80

Garcia M, Talgatti D (2011) Morfologia e distribuição de *Catenula adhaerens* Mereschkowsky (Bacillariophyceae) no sul do Brasil. Iheringia Ser Bot 66: 99–108

Giffen MH (1973) Diatoms of the marine littoral of the Steenberg's Cove in St Helena Bay, Cape Province, South Africa. Bot Mar 16: 32–48

Giffen MH (1975) An account of the littoral diatoms of from Langebaan, Saldahna Bay, Cape Province, South Africa. Bot Mar 18: 71–95

Giffen MH (1976) A further account of the littoral diatoms of the Saldahna Bay Lagoon, Cape Province, South Africa. Bot Mar 19: 379–394

Kaleli MA, Kulikovskiy MS, Solak CN (2017) Some New Records for Marine Diatom Flora of Turkey From Akliman, Sinop (Black Sea) Turk J Fish Aquat Sc 17: 1387–1395

Kaleli A, Krzywda M, Witkowski A, Riaux-Gobin C, Solak CN, Zgłobicka I, Płociński T, Grzonka J, Kurzydłowski KJ, Car A (2018) A new sediment dwelling and epizoic species of *Olifantiella* (Bacillariophyceae), with an account on the genus ultrastructure based on Focused Ion Beam nanocuts. Fottea 18: 212–226

Kim BS, Kim SY, Park J–G, Witkowski A (2017) New Records of the Diatom Species (Bacillariophyta) from the Seaweed and Tidal Flats in Korea. Environ Biol Res 35: 604–621

Lee SS, Gaiser EE, Van de Vijver B, Edlund MB, Spaulding SA (2014) Morphology and typification of *Mastogloia smithii* and *M. lacustris*, with descriptions of two new species from the Florida Everglades and the Caribbean region. Diatom Res 29: 325–350

Lobban CS (2015) A second species of *Microtabella* (Grammatophoraceae, Bacillariophyta) from Guam. Mar Biodivers Rec 8: 1–5

Lobban CS, Ashworth MP (2014) *Hanicella moenia*, gen. et sp. nov., a ribbon–forming diatom (Bacillariophyta) with complex girdle bands, compared to *Microtabella interrupta* and *Rhabdonema* cf. *adriaticum*: Implications for Striatellales, Rhabdonematales, and Grammatophoraceae, fam. nov. J Phycol 50: 860–884

Lobban CS, Ashworth MP, Arai Y, Jordan RW, Theriot EC (2011) Marine necklace-chain Fragilariaceae (Bacillariophyceae) from Guam, including descriptions of *Koernerella* and *Perideraion*, genera nova. Phycol Res 59: 175–193

Lobban CS, Schefter M, Jordan RW, Arai Y, Sasaki A, Theriot EC, Ashworth MP, Ruck EC, Pennesi C (2012) Coral–reef diatoms (Bacillariophyta) from Guam: New records and preliminary

checklist, with emphasis on epiphytic species from farmer–fish territories. Micronesica 43: 237–479

Majewska R, Kociolek P, Thomas E, De Stefano M, Santoro M, Bolaños F, Van de Vijver B (2015b) *Chelonicola* and *Poulinea*, two new gomphonemoid diatom genera (Bacillariophyta) living on marine turtles from Costa Rica. Phytotaxa 233: 236–250

Majewska R, De Stefano M, Ector L, Bolanos F, Frankovich TA, Sullivan MJ, Ashworth MP, Van de Vijver B (2017b) Two new epizoic *Achnanthes* species (Bacillariophyta) living on marine turtles from Costa Rica Bot Mar 60: 303–318

Majewska R, Van de Vijver B, Nasrolahi A, Ehsanpour M, Afkhami M, Bolaños F, Iamunno F, Santoro M, De Stefano M (2017a) Shared epizoic taxa and differences in diatom community structure between green turtles (*Chelonia mydas*) from distant habitats. Microbial Ecol 74: 969–978

Majewska R, Bosak S, Frankovich TA, Ashworth MP, Sullivan MJ, Robinson NJ, Lazo-Wasem EA, Pinou T, Nel R, Manning SR, Van de Vijver B (2019) Six new epibiotic *Proschkinia* (Bacillariophyta) species and new insights into the genus phylogeny Eur J Phycol 54: 609–631

Majewska R, De Stefano M, Van de Vijver B (2017c) *Labellicula lecohuiana*, a new epizoic diatom species living on green turtles in Costa Rica. Nova Hedwigia 146: 23–31

Majewska R, Robert K, Van de Vijver B, Nel R (2019) A new species of *Lucanicum* (Cyclophorales, Bacillariophyta) associated with loggerhead sea turtles from South Africa. Bot Letters (publ. online)

Park J, Khim JS, Ohtsuka T, Araki H, Witkowski A, Koh C–H (2012) Diatom assemblages on Nanaura mudflat, Ariake Sea, Japan: With reference to the biogeography of marine benthic diatoms in Northeast Asia. BotanStud 53: 105–124

Park J, Khim JS, Ryu J, Koh C–H, Witkowski A (2013) An emended description of the genus Fogedia (Bacillariophyceae) with reports of four species new to science from a Korean sand flat. Phycologia 52: 437–446

Park JS, Lobban CS, Lee K–W (2018) Diatoms associated with seaweeds from Moen Island in Chuuk Lagoon, Micronesia. Phytotaxa 351: 101–140

Riaux–Gobin C, Witkowski A, Ruppel M (2012) *Scalariella* a new genus of monoraphid diatom (Bacillariophyta) with a bipolar distribution. Fottea 12: 13–25

Romagnoli T, Totti C, Accoroni S, De Stefano M, Pennesi C (2014) SEM analysis of the epibenthic diatoms on *Eudendrium racemosum* (Hydrozoa) from the Mediterranean Sea. Turk J Bot 38: 1–29

Sar EA, Romero OE, Sunesen I (2003) *Cocconeis* Ehrenberg and *Psammococconeis* Garcia (Bacillariophyta) from the Gulf of San Matías, Patagonia, Argentina. Diatom Res 18: 79–106

Sar EA, Sunesen I, Fernandez PV (2007) Marine Diatoms from Buenos Aires coastal waters (Argentina) II Thalassionemataceae and Rhaphoneidaceae. Rev Chil Hist Natural 80: 63–79

Siqueiros-Beltrones DA, Argumedo-Hernández U, Landa-Cancigno C (2015) Uncommon species diversity values in epiphytic diatom assemblages of the kelp *Eisenia arborea*. Hidrobiol 26: 61–76

Siqueiros-Beltrones DA, Argumedo-Hernández U, López-Fuerte FO (2017) Diversity of benthic diatoms in the Guerrero Negro Lagoon (El Vizcaíno Biosfere Reserve), Baja California Peninsula, Mexico. Rev Mex Biodiver 88: 21–35

Siqueiros-Beltrones DA, Argumedo-Hernández U, Murillo-Jiménez JM, Marmolejo-Rodriguez AJ (2014) Diversidad de diatomeas bentónicas marinas en un ambiente ligeramente enriquecido con elementos potencialmente tóxicos. Rev Mex Biodiver 84: 1065–1085

Siqueiros-Beltrones DA, Hernández-Almeida O (2006) Florística de diatomeas epifitas en un manchón de macroalgas subtropicales. CICIMAR Oceánides 21: 11–61

Siqueiros-Beltrones DA, Martinez, YJ (2017) Prospective floristics of epiphytic diatoms on Rhodophyta from the southern Gulf of Mexico. CICIMAR Oceánides 32: 35–49

Siqueiros-Beltrones DA, Valenzuela-Romero G, Hernández-Almeida O, Argumedo-Hernández U, López-Fuerte FO (2004) Catalogo iconografico de diatomeas de habitats rocosos y su incidencia en la dieta de abulones (Haliotis spp) jovenes de Baja California Sur, México. CICIMAR Oceánides 19: 29–103

Stepanek JG, Kociolek JP (2013) Several new species of *Amphora* and *Halamphora* from the western USA. Diatom Res 28: 61–76

Ulanova A, Snoeijs P (2006) *Lunella garyae* sp nov – a new polyhalobous diatom from the English Channel coast. Diatom Res 21: 415–426

Van de Vijver B, Kociolek JP (2018) A new species of *Nagumoea* (Bacillariophyta) from Antarctica, and a further consideration of the systematic position of the genus. Phytotaxa 349: 152–158

Wachnicka AH, Gaiser EE (2007) Characterization of *Amphora* and *Seminavis* from south Florida, USA. Diatom Res 22: 387–455

Witkowski A, Li C, Zgłobicka I, Yu S, Ashworth MP, Dąbek P, Qin S, Tang C, Krzywda M, Ruppel M, et al (2016) Multigene Assessment of Biodiversity of Diatom(Bacillariophyceae) Assemblages from the Littoral Zone of the Bohai and Yellow Seas in Yantai Region of Northeast China with some Remarks on Ubiquitous Taxa. J Coast Res: Special Issue 74: 166–195

Witkowski A, Metzeltin D, Lange-Bertalot H, Bafana G (1997) *Fogedia* gen nov (Bacillariophyceae), a new naviculoid genus from the marine littoral. Nova Hedwigia 65: 79–98
