# Supplementary material for: Geographical variation in the diatom communities associated with loggerhead sea turtles (Caretta caretta)
Source: PLoS One. 2020 Jul 29;15(7):e0236513. doi: 10.1371/journal.pone.0236513 (PMC7390603; doi:10.1371/journal.pone.0236513)
Supplement: S2 Table — Presumably exclusively epizoic taxa are indicated in bold. (PDF) [file pone.0236513.s002.pdf]

S2 Table List of 397 taxa observed in the carapace and skin samples of the four sampling localities. Presumably exclusively epizoic taxa are indicated in bold.

Taxon	Croatia	Florida	Greece	South Africa
Achnanthes brevipes C.Agardh	x		x	
***Achnanthes elongata* Majewska & Van de Vijver**	x	x	x	x
Achnanthes cf. *danica* (Flögel) Grunow				x
Achnanthes fimbriata (Grunow) R.Ross				x
Achnanthes manifera Brun				x
Achnanthes CRO sp.1	x			x
Achnanthes CRO sp.2	x			x
Achnanthes SA sp.1				x
Achnanthes SA sp.1				x
Achnanthes SA sp.2				x
Achnanthidium GRE sp.1			x	
Actinocyclus ehrenbergii Ralfs		x	x	
Actinocyclus FLO sp.1		x		
Actinocyclus SA sp.1				x
Amphicocconeis cf. *mascarenica* Riaux Gobin & Compère				x
Amphipleura SA sp.1				x
Amphora bigibba var. *interrupta* (Grunow) Cleve				x
Amphora caribaea Wachnicka & E.E.Gaiser		x		
Amphora corpulenta Cleve & Grove		x		
Amphora crenulata A.H.Wachnicka & E.E.Gaiser	x	x	x	x
Amphora gigantea Grunow		x		
Amphora graeffeana Hendey		x		
Amphora obtusa W.Gregory				x
Amphora obtusiuscula Grunow				x
Amphora ostrearia Brébisson ex Kützing			x	
Amphora tenerrima Aleem & Hustedt				x
Amphora cf. *marina* W.Smith			x	
Amphora cf. *hamata* Heiden				x
Amphora cf. *wisei* (M.M.Salah) Simonsen				x
Amphora CRO sp.1	x			x
Amphora CRO sp.2	x			x
Amphora CRO sp.3	x			
Amphora FLO sp.1		x		x
Amphora FLO sp.2		x	x	
Amphora GRE sp.1			x	
Amphora GRE sp.2			x	
Amphora GRE sp.3			x	
Amphora GRE sp.4			x	

Amphora GRE sp.5			x	
Amphora SA sp.1				x
Amphora SA sp.2				x
Anaulus SA sp.1				x
Ardissonea formosa (Hantzsch) Grunow		x		
Astartiella SA sp.1				x
Astartiella SA sp.2				x
Austariella admissa (Hustedt) Witkowski, Lange Bertalot & Metzeltin				x
Berkeleya fennica Juhlin Dannfelt	x		x	
Berkeleya hyalina (Round & M.E.Brooks) E.J.Cox				x
Berkeleya micans (Lyngbye) Grunow	x		x	
Berkeleya scopulorum (Brébisson ex Kützing) E.J.Cox	x			
Biddulphia biddulphiana (J.E.Smith) Boyer	x			
Bifibulatia CRO sp.1				
Brachysira aponina Kützing	x			
Brachysira estonarium Witkowski, Lange Bertalot & Metzeltin		x		
Caloneis excentrica (Grunow) Boyer		x		
Caloneis fusioides (Grunow) Heiden & Kolbe			x	
Caloneis liber (W.Smith) Cleve	x	x	x	x
Caloneis liber var. *linearis* Cleve		x		x
Caloneis FLO sp.1		x		
Campylodiscus cf. *neofastuosus* Ruck & Nakov			x	
Campylodiscus ralfsii W.Smith		x		
Campylodiscus FLO sp.1		x		
Campyloneis SA sp.1				x
Carinasigma GRE sp.1			x	
Catenula exigua K.Robert, Bosak & Van de Vijver	x			
***Chelonicola* SA sp.1**				x
Climaconeis inflexa (Brébisson ex Kützing) E.J.Cox				x
Cocconeis convexa M.H.Giffen	x			x
Cocconeis distans W.Gregory				x
Cocconeis lineata Ehrenberg	x	x	x	x
Cocconeis maxima (Grunow) H.Peragallo & M.Peragallo			x	
Cocconeis molesta Kützing				x
Cocconeis pinnata W.Gregory ex Greville				x
Cocconeis scutellum Ehrenberg var. 1			x	
Cocconeis scutellum Ehrenberg var. 2			x	x
Cocconeis scutellum Ehrenberg var. 3	x		x	x
Cocconeis cf. *distantula* M.H.Giffen				x
Cocconeis cf. *dirupta* W.Gregory				x

Cocconeis cf. *pediculus* Ehrenberg	x			
Cocconeis cf. *peltoides* Hustedt				x
Cocconeis cf. *sigillata* Riaux Gobin & Al Handal				x
Cocconeis CRO sp.1	x			
Cocconeis CRO sp.2	x			
Cocconeis CRO sp.3	x			
Cocconeis CRO sp.4	x			
Cocconeis FLO sp.1		x		
Cocconeis GRE sp.1			x	
Cocconeis SA sp.1				x
Cocconeis SA sp.2				x
Cocconeis SA sp.3				x
Cocconeis SA sp.4				x
Cocconeis SA sp.5				x
Coscinodiscus GRE sp.1			x	
Coscinodiscus SA sp.1				x
***Craspedostauros* SA sp.1**				x
***Craspedostauros* CRO sp.1**	x			
Cyclophora tenuis Castracane	x			x
Cyclophora tabellariformis Ashworth & Lobban				x
Cyclotella litoralis Lange & Syvertsen		x		
Cylindrotheca closterium (Ehrenberg) Reimann & J.C.Lewin			x	
Cymatosira cf. *lorenziana* Grunow		x		
Cymatosira SA sp.1				x
Cymatosira SA sp.2				x
Cymbella hustedtii Krasske			x	
Delphineis cf. *minutissima* (Hustedt) Simonsen	x			x
Delphineis SA sp.1				x
Dickieia GRE sp.1			x	
Dimeregramma minor (Gregory) Ralfs in Pritchard	x		x	
Diploneis bombus (Ehrenberg) Ehrenberg			x	
Diploneis suborbicularis (W.Gregory) Cleve		x		
Diploneis cf. *notabilis* (Greville) Cleve			x	
Diploneis cf. *sejuncta* (A.W.F.Schmidt) E.Jørgensen	x			
Diploneis CRO sp.1	x			
Diploneis CRO sp.2	x	x	x	x
Diploneis CRO sp.3	x			
Diploneis CRO sp.4	x			
Diploneis CRO sp.5	x			
Diploneis CRO sp.6	x			
Diploneis CRO sp.7	x			
Diploneis CRO sp.8	x			
Diploneis CRO sp.9	x			
Diploneis GRE sp.1			x	

Diploneis GRE sp.2			x	
Diploneis SA sp.1				x
Diploneis SA sp.2				x
Diploneis SA sp.3				x
Diploneis SA sp.4				x
Diploneis SA sp.5				x
Discostella CRO sp.1	x		x	
Discostella GRE sp.1			x	
Encyonema CRO sp.1	x			
Encyonema minutum (Hilse) D.G.Mann	x			
Epithemia constricta Brébisson			x	
Epithemia musculus Kützing	x			
Epithemia pacifica (Krammer) Lobban & J.S.Park		x		
Epithemia SA sp.1				x
Falcula SA sp.1				x
Fallacia CRO sp.1	x		x	x
Fallacia CRO sp.2	x			x
Fallacia forcipata (Greville) Stickle & D.G.Mann			x	
Fallacia schoemaniana (Foged) Witkowski				x
Fallacia subforcipata (Hustedt) D.G.Mann			x	
Fallacia SA sp.1				x
Fogedia giffeniana (Foged) Witkowski, Lange Bertalot, Metzeltin & Bafana			x	
Fogedia heterovalvata Witkowski, Metzeltin & Lange Bertalot				x
Fragilaria CRO sp.1	x			
Fragilaria FLO sp.1		x		
Fragilariopsis SA sp.1				x
Glyphodesmis cf. *distans* (W.Gregory) Grunow	x			
Gomphonema GRE sp.1			x	
Gomphonema micropus Kützing			x	
Gomphonemopsis pseudexigua Medlin	x		x	
Grammatophora cf. *marina* (Lyngbye) Kützing			x	
Grammatophora CRO sp.1	x			
Grammatophora FLO sp.1		x		
Grammatophora FLO sp.2		x		
Grammatophora FLO sp.3		x	x	
Grammatophora GRE sp.1			x	
Grammatophora undulata Ehrenberg				x
Gyrosigma tenuissimum (W.Smith) J.W.Griffith & Henfrey				x
Gyrosigma GRE sp.1			x	
Halamphora kolbei (Aleem) Álvarez Blanco & S.Blanco	x		x	x
Halamphora cf. *cejudoae* Álvarez Blanco & S.Blanco	x		x	x

Halamphora cf. *coffeaeformis* (C.Agardh) Levkov			x	x
Halamphora cf. *holsatica* (Hustedt) Levkov			x	x
Halamphora cf. *turgida* (Gregory) Levkov				x
Haslea GRE sp.1			x	
Haslea SA sp.1				x
Haslea nautica (Cholnoky) Giffen				x
Hippodonta linearis (Østrup) Lange Bertalot, Metzeltin & Witkowski				x
Hyalosira hesperia Álvarez Blanco & S.Blanco	x		x	x
Hyalosira tropicalis J.N.Navarro				x
Hyalosira SA sp.1				x
Hyalosynedra hyalina (Grunow) Álvarez Blanco & S.Blanco	x	x		x
Hyalosynedra laevigata (Grunow) D.M.Williams & Round	x	x	x	x
Hyalosynedra lanceolata Belando, Jiménez & Aboal	x	x		
Hyalosynedra CRO sp. 1	x			x
Karayevia ploenensis (Hustedt) Bukhtiyarova			x	
Koernerella recticostata (Körner) M.P.Ashworth, C.S.Lobban & E.C.Theriot				x
Kolbesia aff. *sinica* Krzywda, Witkowski & Ch.Li	x			
Lancineis fatula (K.E.Lohman) G.W.Andrews				x
Licmophora abbreviata C.Agardh	x			
Licmophora debilis (Kützing) Grunow	x		x	
Licmophora ehrenbergii (Kützing) Grunow				x
Licmophora flabellata (Greville) C.Agardh			x	x
Licmophora gracilis (Ehrenberg) Grunow				x
Licmophora hyalina (Kützing) Grunow	x			
Licmophora paradoxa (Lyngbye) C.Agardh			x	x
Licmophora proboscidea Mereschkowsky	x		x	
Licmophora remulus (Grunow) Grunow			x	x
Licmophora cf. *curvata* Lobban, Tharngan & Ashworth				x
Licmophora cf. *flucticulata* Lobban, Schefter & Ruck				x
Licmophora cf. *gracilis* (Ehrenberg) Grunow				x
Licmophora cf. *paradoxa* (Lyngbye) C.Agardh				x
Licmophora CRO sp.1	x			
Licmophora SA sp. 1				x
Licmophora SA sp. 2				x
Licmophora SA sp. 3				x
Licmophora SA sp. 4				x
Licmophora SA sp. 5				x
Licmophora SA sp. 6				x

Lucanicum ashworthianum Majewska, K.Robert & Van de Vijver				x
Lyrella cf. *abrupta* (W.Gregory) D.G.Mann			x	
Lyrella cf. *lyroides* (Hendey) D.G.Mann			x	
Mastogloia angulata F.W.Lewis	x	x		
Mastogloia apiculata W.Smith				x
Mastogloia bahamensis Cleve		x		
Mastogloia binotata (Grunow) Cleve	x	x		x
Mastogloia biocellata (Grunow) G.Novarino & A.R.Muftah		x	x	
Mastogloia corsicana Grunow		x		x
Mastogloia cribrosa Grunow		x		
Mastogloia crucicula (Grunow) Cleve	x	x	x	x
Mastogloia cuneata (Meister) Simonsen	x		x	x
Mastogloia cyclops Voigt				x
Mastogloia depressa Hustedt		x		
Mastogloia elegans Lewis		x		
Mastogloia emarginata Hustedt		x		x
Mastogloia erythraea Grunow		x		
Mastogloia erythraea var. *grunowii* Foged		x		x
Mastogloia fimbriata (T.Brightwell) Grunow		x		
Mastogloia graciloides Hustedt	x			
Mastogloia horvathiana Grunow		x		
Mastogloia ignorata Hustedt			x	
Mastogloia lacrimata Voigt		x		
Mastogloia manokwariensis Cholnoky	x		x	
Mastogloia ovalis A.Schmidt				x
Mastogloia paradoxa Grunow			x	
Mastogloia pseudolacrimata T.A.Yohn & R.A.Gibson		x		
Mastogloia pumila (Grunow) Cleve	x		x	
Mastogloia punctifera Brun		x		
Mastogloia pusilla Grunow	x	x	x	
Mastogloia robusta Hustedt		x	x	
Mastogloia aff. *ovalis* A.Schmidt	x	x	x	x
Mastogloia cf. *acutiuscula* Grunow	x			
Mastogloia cf. *grunowii* A.Schmidt			x	
Mastogloia cf. *pusilla* Grunow			x	
Mastogloia CRO sp.1	x			
Mastogloia CRO sp.2	x			
Mastogloia FLO sp.1		x		
Mastogloia FLO sp.2		x		
Mastogloia FLO sp.3		x		
Mastogloia FLO sp.4		x		
Mastogloia FLO sp.5		x		
Mastogloia FLO sp.6		x		

Mastogloia SA sp.1				x
Mastogloia SA sp.2				x
***Medlinella amphoroidea* Frankovich, Ashworth & M.J. Sullivan**			x	
Melosira moniliformis C.Agardh	x		x	
Microtabella interrupta (Ehrenberg) Round		x		
Microtabella cf. *delicatula* (Kützing) Round	x	x	x	x
Nagumoea CRO sp. 1	x			
Nanofrustulum shiloi (J.J.Lee, Reimer & McEnery) Round, Hallsteinsen & Paasche	x		x	
Navicula agatkae Witkowski			x	
Navicula directa (W.Smith) Ralfs				x
Navicula gregaria Donkin			x	
Navicula cf. *cancellata* Donkin			x	x
Navicula cf. *directa*		x	x	
Navicula cf. *kariana* Grunow	x			
Navicula cf. *korzeniewskii* Witkowski	x			
Navicula cf. *pavillardii* Hustedt			x	x
Navicula cf. *perminuta* Grunow	x		x	x
Navicula cf. *phyllepta* Kützing			x	
Navicula cf. *subagnita* Proshkina Lavrenko			x	
Navicula CRO sp.1	x	x		x
Navicula CRO sp.2	x			
Navicula CRO sp.3	x			
Navicula CRO sp.4	x			
Navicula CRO sp.5	x			
Navicula CRO sp.6	x			
Navicula CRO sp.7	x	x	x	
Navicula CRO sp.8	x			
Navicula FLO sp.1		x		
Navicula FLO sp.2		x		
Navicula FLO sp.3		x		
Navicula GRE sp.1			x	
Navicula GRE sp.2			x	
Navicula GRE sp.3			x	
Navicula GRE sp.4			x	
Navicula SA sp.1				x
Navicula SA sp.2				x
Navicula SA sp.3				x
Navicula SA sp.4				x
Navicula SA sp.5				x
Navicula SA sp.6				x
Neosynedra provincialis (Grunow) D.M.Williams & Round	x			x
Neosynedra tortuosa (Grunow) D.M.Williams & Round				x
Nitzschia angularis W.Smith		x		

Nitzschia amabilis H.Suzuki	x		x	x
Nitzschia dissipata (Kützing) Rabenhorst			x	
Nitzschia scalpelliformis Grunow	x	x	x	x
Nitzschia sicula (Castracane) Hustedt	x			
Nitzschia sigma (Kützing) W.Smith			x	
Nitzschia spathulata Brébisson ex W.Smith			x	x
Nitzschia valdestriata Aleem & Hustedt	x			
Nitzschia ventricosa Kitton				x
Nitzschia cf. *acicularis* (Kützing) W.Smith				x
Nitzschia cf. *bremensis* Hustedt	x			
Nitzschia cf. *inconspicua* Grunow	x		x	x
Nitzschia cf. *longissima* (Brébisson) Ralfs	x	x		
Nitzschia cf. *agnita* Hustedt				x
Nitzschia cf. *angustatula* Lange Bertalot				x
Nitzschia CRO sp.1	x			
Nitzschia CRO sp.2	x	x	x	x
Nitzschia CRO sp.3	x			
Nitzschia CRO sp.4	x			
Nitzschia CRO sp.5	x			
Nitzschia CRO sp.6	x			
Nitzschia FLO sp.1		x		
Nitzschia FLO sp.2		x		
Nitzschia FLO sp.3		x		
Nitzschia GRE sp.1			x	
Nitzschia GRE sp.2			x	
Nitzschia GRE sp.3		x	x	
Nitzschia SA sp.1				x
Nitzschia SA sp.2				x
Nitzschia SA sp.3				x
Odontella CRO sp.1	x			
Oestrupia grandis M.K.Hein & B.M.Winsborough		x		
Olifantiella cf. *mascarenica* Riaux Gobin & Compère	x		x	
Opephora pacifica (Grunow) Petit			x	
Opephora GRE sp.1			x	
Opephora GRE sp.2			x	
Opephora GRE sp.3			x	
Pantocsekiella ocellata (Pantocsek) K.T.Kiss & Ács			x	
Paralia sulcata (Ehrenberg) Cleve	x		x	
Parlibellus berkeleyi (Kützing) E.J.Cox	x			
Parlibellus SA sp.1				x
Parlibellus SA sp.2				x
Pauliella cf. *taeniata* (Grunow) Round & Basson				x
Pauliella taeniata (Grunow) Round & Basson				x
Plagiodiscus nervatus Grunow				x

Plagiotropis lepidoptera var. *proboscidea* (Cleve) Reimer		x		
Plagiotropis SA sp. 1				x
Planothidium kaetherobertianum Van de Vijver & Bosak	x			
Planothidium GRE sp.1			x	
Planothidium GRE sp.2			x	
Planothidium SA sp.1				x
Planothidium SA sp.2				x
Pleurosigma GRE sp.1			x	
Podocystis spathulata (Shadbolt) Van Heurck				x
***Poulinea* CRO sp.1**	x		x	
***Poulinea* CRO sp.2**	x	x	x	
***Proschkinia sulcata* Majewska, Van de Vijver & Bosak**	x			
***Proschkinia vergostriata* Frankovich, Ashworth & M.J.Sullivan**	x		x	x
Proschkinia GRE sp.1			x	x
Proschkinia SA sp.1				x
Psammodictyon coarctata (Grunow) Mann	x	x	x	x
Psammodictyon cf. *panduriforme* (W.Gregory) D.G.Mann	x			x
Psammodictyon SA sp. 1				x
Psammodiscus cf. *nitidus* (W.Gregory) Round & D.G.Mann	x			
Pseudostaurosira GRE sp. 1			x	
Rhabdonema adriaticum Kützing			x	
Rhaphoneis capensis A.W.F.Schmidt				x
Rhopalodia acuminata Krammer	x		x	
Seminavis basilica Danielidis	x			
Seminavis cyrtorapha A.H.Wachnicka & E.E.Gaiser		x		
Seminavis delicatula Wachnicka & Gaiser				x
Seminavis insignis Álvarez Blanco & S.Blanco			x	
Seminavis robusta D.B.Danielidis & D.G.Mann			x	
Seminavis strigosa (Hustedt) Danieledis & Economou Amilli	x	x	x	
Seminavis cf. *delicatula* Wachnicka & Gaiser		x		x
Seminavis cf. *obtusiuscula* (Grunow) Danieledis & D.G.Mann				x
Seminavis GRE sp.1			x	
Seminavis GRE sp.2			x	
Seminavis SA sp.1				x
Seminavis SA sp.2				x
Stauronella indubitabilis Lange Bertalot & S.I.Genkal			x	
Staurosiralla SA sp.1				x

Striatella unipunctata (Lyngbye) C.Agardh	x			x
Surirella CRO sp.1	x			
Synedra bacillaris (Grunow) Hustedt		x		
Synedra commutata Grunow	x			
Synedra cf. *fulgens* (Greville) W.Smith				x
Synedrosphenia FLO sp. 1		x		
Tabularia affinis (Kützing) Snoeijs		x		
Tabularia fasciculata (C.Agardh) D.M.Williams & Round			x	x
Tabularia tabulata (C.Agardh) Snoeijs			x	
Tabularia cf. *investiens* (W.Smith) D.M.Williams & Round	x		x	x
Tabularia cf. *waernii* P.Snoeijs				x
Tabularia CRO sp.1	x			
Tabularia FLO sp.1		x		
Thalassionema frauenfeldii (Grunow) Tempère & Peragallo	x		x	
Thalassiosira CRO sp. 1	x			x
Toxarium hennedyanum (Gregory) Pelletan		x		
Toxarium undulatum Bailey	x			
Trachyneis aspera (Ehrenberg) Cleve				x
Trachysphenia australis P.Petit				x
Trachysphenia FLO sp.1		x		
Tryblionella compressa (Bailey) Poulin	x		x	
Tryblionella CRO sp.1	x			
Tryblionella granulata (Grunow) D.G.Mann			x	
Tryblionella lanceola Grunow			x	
Tryblionella pararostrata (Lange Bertalot) Clavero & Hernández Mariné			x	
Tryblionella SA sp.1				x
Tryblionella apiculata W.Gregory			x	
Tryblionella persuadens (Cholnoki) K.P.Cavalcante, P.I.Tremarin & T.A.V.Ludwig			x	
Ulnaria delicatissima (W.Smith) Aboal & P.C.Silva			x	
