# Supplementary material for: Geographical variation in the diatom communities associated with loggerhead sea turtles (Caretta caretta)
Source: PLoS One. 2020 Jul 29;15(7):e0236513. doi: 10.1371/journal.pone.0236513 (PMC7390603; doi:10.1371/journal.pone.0236513)
Supplement: S3 Table — SIMPER analysis was based on Bray-Curtis similarity, 70% cut off, taxa cumulatively contributing to the similarity over 70% are shown. Croatia (CRO), Greece (GRE), South Africa (SA), Florida (FLO). (PDF) [file pone.0236513.s003.pdf]

**S3 Table similarity analysis of loggerhead epizoic diatom assemblages within each sampling locality – typical species.** SIMPER analysis was based on Bray-Curtis similarity, 70% cut off, taxa cumulatively contributing to the similarity over 70% are shown. Croatia (CRO), Greece (GRE), South Africa (SA), Florida (FLO)

| Species | Average Abundance | Average Simmilarity | Dissimilarity /SD | Contributio n % | Cumulative contribution |
|---|---|---|---|---|---|
| **Croatia - average similarity = 21,05** | | | | | |
| *Poulinea* CRO sp.1 | 3,71 | 3,44 | 0,98 | 16,34 | 16,34 |
| *Poulinea* CRO sp.2 | 5,37 | 2,71 | 0,98 | 12,87 | 29,21 |
| *Amphora crenulata* | 3,54 | 2,62 | 0,96 | 12,45 | 41,66 |
| *Nitzschia* CRO sp.2 | 3,89 | 1,72 | 0,63 | 8,18 | 49,84 |
| *Neosynedra provincialis* | 1,97 | 1,51 | 0,62 | 7,16 | 57 |
| *Berkeleya fennica* | 3,24 | 1,44 | 0,62 | 6,84 | 63,83 |
| *Licmophora debilis* | 1,33 | 1,03 | 0,6 | 4,88 | 68,71 |
| *Tabularia* cf. *investiens* | 1,46 | 0,9 | 1,15 | 4,27 | 72,98 |
| *Hyalosira hesperia* | 1,52 | 0,73 | 0,62 | 3,47 | 76,45 |
| **Florida - average similarity= 60,36** | | | | | |
| *Hyalosynedra laevigata* | 17,56 | 45,6 | 6,11 | 75,56 | 75,56 |
| **Greece carapace - average similarity: 57,83** | | | | | |
| *Nitzschia* cf. *incospicua* | 9,61 | 10,22 | 1,8 | 17,68 | 17,68 |
| *Nitzschia* CRO sp.2 | 7,38 | 8,65 | 3,9 | 14,96 | 32,63 |
| *Cocconeis lineata* | 6,45 | 8,23 | 5,79 | 14,24 | 46,87 |
| *Navicula* cf. *pavilardii* | 4,29 | 5,78 | 6 | 10 | 56,87 |
| *Navicula* cf. *perminuta* | 4,29 | 4,68 | 4,58 | 8,09 | 64,96 |
| *Amphora crenulata* | 4,36 | 4,36 | 2,92 | 7,54 | 72,5 |
| *Seminavis insignis* | 2,18 | 1,99 | 2,32 | 3,44 | 75,94 |
| **Greece skin - average similarity: 62,11** | | | | | |
| *Nitzschia* CRO sp.2 | 10,46 | 15,46 | 2,72 | 24,89 | 24,89 |
| *Navicula* GRE sp.2 | 6,64 | 11,04 | 12,95 | 17,77 | 42,66 |
| *Medlinella amphoroidea* | 8 | 10,31 | 2,48 | 16,6 | 59,26 |
| *Proschkinia* CRO sp.2 | 6,42 | 8,04 | 1,79 | 12,94 | 72,2 |
| **South Africa - average similarity = 49,32** | | | | | |
| *Chelonicola* SA sp. 1 | 13,46 | 25,35 | 3,45 | 51,4 | 51,4 |
| *Nitzschia* cf. *incospicua* | 9,05 | 12,86 | 1,12 | 26,07 | 77,47 |
