# Supplementary material for: Geographical variation in the diatom communities associated with loggerhead sea turtles (Caretta caretta)
Source: PLoS One. 2020 Jul 29;15(7):e0236513. doi: 10.1371/journal.pone.0236513 (PMC7390603; doi:10.1371/journal.pone.0236513)
Supplement: Attachment — Submitted filename: Response to Reviewers_Van de Vijver.docx [file pone.0236513.s004.docx]

PONE-D-20-05852
Diversity of diatom communities associated with loggerhead sea turtles (*Carreta carreta*)
PLOS ONE

**Response to reviewers**

Dear Editor,

Thank you for the consideration of our manuscript for the publication in PLOS ONE. The authors thank the two reviewers for their thorough review, comments, and suggestions. We believe that the revisions substantially improved the manuscript. We made the requested changes, both in the text and in the figures, please find our answer (A) to the journal requirements (J) and reviewers’ suggestions (R) below.

**J:** 1. Please ensure that your manuscript meets PLOS ONE's style requirements, including those for file naming. The PLOS ONE style templates can be found at <http://www.plosone.org/attachments/PLOSOne_formatting_sample_main_body.pdf> and <http://www.plosone.org/attachments/PLOSOne_formatting_sample_title_authors_affiliations.pdf>

**A:** Checked and changed accordingly

**J:** 2. Please include a copy of Table 5 which you refer to in your text on page 17.

**A:** The Table caption is corrected to 5.

**J**: 3. We note that Figure 1 in your submission contain map images which may be copyrighted. All PLOS content is published under the Creative Commons Attribution License (CC BY 4.0), which means that the manuscript, images, and Supporting Information files will be freely available online, and any third party is permitted to access, download, copy, distribute, and use these materials in any way, even commercially, with proper attribution. For these reasons, we cannot publish previously copyrighted maps or satellite images created using proprietary data, such as Google software (Google Maps, Street View, and Earth). For more information, see our copyright guidelines: <http://journals.plos.org/plosone/s/licenses-and-copyright>. We require you to either (1) present written permission from the copyright holder to publish these figures specifically under the CC BY 4.0 license, or (2) remove the figures from your submission:

 2. If you are unable to obtain permission from the original copyright holder to publish these figures under the CC BY 4.0 license or if the copyright holder’s requirements are incompatible with the CC BY 4.0 license, please either i) remove the figure or ii) supply a replacement figure that complies with the CC BY 4.0 license. Please check copyright information on all replacement figures and update the figure caption with source information. If applicable, please specify in the figure caption text when a figure is similar but not identical to the original image and is therefore for illustrative purposes only.

**A:** The figure 1. has been replaced with the figure that complies with CC BY 4.0 license according to the recommendation from the Editors office. The caption now says:

**“Fig 1. The sampling areas of loggerhead sea turtles.** (A) Red dots indicate locations of sampled loggerheads. Inserts show details of the sampling locations: (B) Amvrakikos Gulf, Greece; (C) Adriatic Sea, Croatia; (D) Florida Bay, USA; (E) Kosi Bay, South Africa. The maps were made with Natural Earth. Free vector and raster map data @ naturalearthdata.com.”

**Reviewer #1:**

**R:** Van de Vijver et al. present a well-written, interesting, and valuable study. My only major comment is that I believe that at the end of the discussion, I think the paper would benefit from having some more discussion about how the findings of this study could applied. The authors do a great job of explaining the why they think that diatom community structures differ between turtles from different regions; however, they do not really expand on the bigger picture implications of this. I think a rather simple fix would be to acknowledge that as diatom communities may reflect foraging patterns of these turtles, then diatom communities could be used as behavioral indicators for these species.

**A:** Although we appreciate the reviewer’s kind words and suggestions, we believe that if we include the conclusion proposed by the reviewer, we should have observed behavior and foraging patterns of the sea turtles that we investigated, and this was not done in our study. Also, this paper includes only one sea turtle species and we think more detailed research including comparisons between different host species with different habitats and behaviors is needed. The main objective of this study was to provide a baseline for the future epizoic diatom studies on loggerheads and we do not want to speculate about bigger implications until they can be proven. However, we have included the following sentences at the end of the abstract that we feel are appropriate conclusions drawn from the results of this study.

*“Our results indicate that epizoic diatom communities differ according to host species geographical location and host substrate (skin vs. carapace). The relative abundances of common benthic diatoms and putative exclusive epizoic taxa may inform about host habitat use or behavior though detailed comparisons between different host sea turtle species have yet to be performed.”*

**R:** All other comments are relatively minor and are listed below:
Line 25: “Epizoic diatoms form an important part of micro-epibiota on marine vertebrates” should be “Epizoic diatoms form an important part of the micro-epibiota of marine vertebrates”.

**A**: Corrected

**R:** Line 29 - 30: “Almost 400 diatom taxa belonging to more than 100 genera have been observed.” Because this sentence is written in the passive tense, it is not clear whether you are referring to only the diatom taxa observed in this study or all the taxa reported in all the published literature on this subject. I would switch this sentence to the active tense. I.e. “ We observed almost 400 diatom taxa belonging to more than 100 genera.” There are also several other examples in the document where it would be clearer to use the active tense instead of the passive tense. Please change throughout.

**A:** We have changed suggested use from passive voice to active, as the reviewer suggested.

**R:** Line 31: “Diatom communities from Greece and Croatia showed the highest similarity, clearly differing from the communities observed in the samples from South Africa and Florida.” should be “Diatom communities from Greece and Croatia showed the highest similarity and were statistically different to those recorded from South Africa and Florida.”

**A**: Corrected

**R:** Line 32: “Part of the difference in diversity and composition may be attributed to different sampling techniques.” I think this could be rephrased as, at present, it largely undermined the entire study. How about “While sampling technique different between locations, and this may affect some of various we observed between localities, we believe that geography played a larger role”.

**A**: Thank you for this comment, we have changed this sentence according to your suggestion.

**R:** Line 38: The abstract would strongly benefit from a concluding sentence that explains what are the implications of your findings.

**A**: We added the following sentences:

*“Our results indicate that epizoic diatom communities differ according to host species geographical location and host substrate (skin vs. carapace). The relative abundances of common benthic diatoms and putative exclusive epizoic taxa may inform about host habitat use or behavior though detailed comparisons between different host sea turtle species have yet to be performed.”*

**R:** Line 46: “everywhere where” should be “wherever”.

**A**: Corrected

**R:** Line 416: “Has” should be “have”.

**A**: Corrected

**R:** Line 429: The authors appear to have cited the wrong study here. The authors state “Robinson et al. [14] observed that the macro-epibiont diversity of nesting sea turtles is partially linked to the diversity of their foraging habitats.” However, they cite another study by this author that looks at diatom and noi macro-epibiont diversity. I believe the authors were aiming to cite this study instead “Robinson NJ, Lazo-Wasem EA, Paladino FV, Zardus JD, Pinou T (2017) Assortative epibiosis of leatherback, olive ridley and green sea turtles in the Eastern Tropical Pacific. Journal of the Marine Biological Association of the United Kingdom 97(6):1233-40.”.

**A**: Yes, thank you for this observation, we changed the citation accordingly.

**R:** Reviewer #2: The manuscript is very well written and the graphs are all very clear. Great effort was taken to identify the taxa and the number of taxa found is quite impressive! The abstract provides a nice description of the manuscript.

**A**: Thank you.

**R:** The title could be a bit more descriptive – something like ‘Geographical variation in the diverse diatom communities associated …’.

**A**: The title is changed to:

“Geographical variation in the diatom communities associated with loggerhead sea turtles (Carreta carreta)”

**R:** The goals of the study are simply to document the diatom communities on the turtles and see whether different areas of the turtles have different diatom composition. The manuscript would be greatly improved with a hypothesis to test. For example, although a previous study demonstrated that turtles of different species in the same area have similar diatom epibionts, but you might anticipate differences among turtles from different areas, if these turtles are truly geographically separated. Also, why might you expect skin and carapace areas to have different diatom populations?

**A**: Thank you for your comment, however, as we have stated at the end of our introduction, the objective of the present study was to provide a baseline data for the studies of diatom diversity on loggerheads, therefore the hypothesis such as suggested could not been put forward. We expect exactly to do just that in our following manuscripts, investigate differences and links between different host species and their habitats. The different flora on the skin and carapace was expected due to the characteristics of the substratum as we have explained in the discussion, the skin is a physiologically more active substratum and favors the development of epizoic taxa.

**R:** In the Discussion, there seems to be an ‘all or none’ sort of definition to epizoic taxa -as if they should never be found on non-animal surfaces. Benthic and even attached diatoms often become ‘planktonic’ – at least until they settle on a surface. If epizoic species thereby end up on non-animal sources and survive, this may not mean that they are not epizoic. Especially as the processing method does not allow distinguishing between empty frustules and live diatoms that are not thriving. Similarly, I would expect many benthic diatoms to do well on the carapace (which is relatively inert). The skin is more interesting, as I suspect there is more sloughing and secretions from the turtle – and so it is not surprising that more presumed epizoic taxa were found on the skin samples.

**A**: Yes, it is true, we cannot know for a fact that taxa we found as epizoic cannot survive on other non-animal surfaces, however, to our knowledge, they have not been found yet there, and apparently, these species are developing large populations on animal surfaces, therefore we label them as epizoic.

**R:** Line 64. ‘accidently’ is probably the wrong word. Maybe ‘haphazardly’ might be a better choice.

**A**: Thank you, the word has been replaced

**R:** Lines 64-65. I suspect that true planktonic and tychoplanktonic species may also settle on turtles (no contact with solid surfaces needed).

**A:** Yes, that is true, as the true planktonic species such as *Chaetoceros* and *Pseudonitzschia* can settle (resting stages, vegetative cells) on the marine sediment bottom, especially in the bloom period, they can also be found in samples scraped from sea turtles as they get caught on these animals (as a sediment trap) instead of travelling all the way to the bottom. However, in the sample list that we analyzed as a part of this study, we did not find any examples of these true planktonic taxa.

**R:** Line 97. Are the Adriatic Sea and Amvrakikos Gulf turtles from different subpopulations? (The locations are fairly close and the species is ‘highly migratory’ (line 83). This is mentioned in the Discussion but should be added here.

**A:** Yes, we added this information as reviewer suggested.

**R:** Line 217. Delete the word ‘up’.

**A:** Deleted

**R:** Table 2. In the legend, ‘Number of diatom genera’, should be ‘Number of diatom taxa in the most diverse genera’.

**A:** Yes, corrected.

**R:** Lines 243-244. ‘most frequently occurring’ could mean either having the highest total count or occurring in the most samples or at the most sites. Suggestion: for the first % listed, include the units (percent of what?).

**A:** We have included: *Nitzschia* CRO sp.2 (present 83.3% of all samples)

**R:** Lines 269-270. Maybe drop the color references, as they all look purple – and (I think) this blue vs purple refers only to the symbols in the headings and not the colors used to indicate abundance – and symbol shape is enough. **R:** Line 272. ‘the fill cells the largest abundance’. The ‘largest abundance in the matrix’ can only be one cell – and all the cells are filled (whether by while or by color). Reword.

**A:** We have corrected the Figure caption following both comments:

“**Fig. 3. The most abundant diatom taxa associated with loggerhead sea turtles**

Shade plot illustrating the 25 most abundant taxa recorded on loggerhead carapaces (triangle) and skins (square) from investigated localities based on square root-transformed abundance data. The white cells represent the absence of the taxa and the darkest cells the largest abundances.. Taxa are ordered by a hierarchical cluster analysis of their mutual associations across samples based on Index of Association calculated on the standardized counts. CRO = Croatia, Adriatic Sea; FLO = Florida Bay, USA; GRE = Greece, Amvrakikos Gulf; SA = South Africa, Kosi Bay”

**R:** Line 254. Replace ‘the latter’ with ‘Florida samples’

**A:** Yes, corrected.

**R:** Lines 281-282. This excludes the GRE skin samples, which had the lowest median value.

**A:** Yes, we added at the beginning of the sentence the clarification that it implies only the samples from the carapace.

**R:** Line 305. ‘were the most diverse’ was a bit tricky to decipher, as it wasn’t obvious that the comparison was among the samples within each site. Maybe ‘had the lowest within-site similarity’.

**A:** Thank you, corrected.

**R:** Line 334. There are 5 groups, one of which is a single sample (I don’t think I would call it an outlier).
Also Line 337. ‘with a main group of 3 samples, a single group comprised of one sample, and…

**A:** Thank you, corrected.

**R:** Fig. 5B. If your skin samples were numbered as pairs with the carapace samples, the matching carapace sample to the skin sample within the carapace cluster (GRE-04) would be GRE-03 – which are the 2 farthest apart samples in the cluster. If this is correct, it would be worth noting (especially since the location of GRE-04 within the carapace cluster wasn’t noted).

**A:** We noted the position of GRE-04 in the discussion and offered a possible explanation:

“Skin sample GRE-04 and the matching carapace sample GRE-03 were collected from the same turtle. The high abundances of Nitzschia cf. inconspicua and Navicula sp.7. (Fig 3.) present in above-mentioned skin sample resulted in its grouping with carapace samples.”

**R:** Line 359. Sampling only 5 individuals per species also limits the number of species found (as indicated by the relatively low similarity among individuals within some of the sites).

**A:** Yes, we agree, the following sentence was added to the discussion.

“This number is most likely an underestimation of the exact richness as sampling of a limited number of turtle individuals may limit the number of diatom taxa found.”

**R:** Line 474. The diatom floras might also indicate segregation among populations of sea turtles (especially benthic feeding species).

**A:** We agree as we wrote in our discussion: “Thus, the behavior of the turtles and its impact on the attached diatom flora may explain why a clear bioregionalism was found in the present study. “
