# Supplementary material for: Geographical variation in the diatom communities associated with loggerhead sea turtles (Caretta caretta)
Source: PLoS One. 2020 Jul 29;15(7):e0236513. doi: 10.1371/journal.pone.0236513 (PMC7390603; doi:10.1371/journal.pone.0236513)
Supplement: Attachment — Submitted filename: Review Of Diatoms.docx [file pone.0236513.s005.docx]

Review Of

**Geographical variation in the diatom communities associated with loggerhead sea turtles (Carreta carreta)**

I still agree that the study presented by Van de Vijver et al. is interesting and worthy of publication. Nevertheless, on a detailed read through, I have noted several typos, errors, and situations were the language could be improved. Thus, I think that the text needs some considerable improvements to be at the level where it would be ready for publication. I have listed several comments below. On top of this, I would also recommend that the authors also take the time to give this manuscript a detailed read through before resubmission to make sure that the text is as clear and understandable as possible.

Title: “*Carreta caretta*” should be “*caretta caretta*”.

Line 25: I would delete this first sentence. The second sentence is a perfect location for this manuscript to start.

Line 28: “skins and shells” do not need to be pluralized in this context.

Line 28: Please include the scientific names after you first provide the species common name.

Line 33: “While sampling technique differed between locations, and this may affect some of variations we observed between localities, we believe that geography played a larger role” should be “We believe that part of this variation was due to differences in sampling techniques; however, we still believe that geography had an important role.”

Line 35 – 37: “Only 5 000 of the known diatom species are considered to be marine with 50 another 50 000 diatom species still to be discovered and described in the marine realm” should be “Of these, around 55,000 are estimated to exist in marine habitats. To date however, less than 5,000 of marine diatoms have been described.”

Line 51-52: This sentence should be moved to be the second sentence in this paragraph.

Line 62: You already mention that diatoms are found on whales and sea turtles in Line 57. Thus, it is a little confusing to again mention all the different species that diatoms are found on just a few sentences later. I would delete Lines 62 – 65 and start by talking about sea turtles.

Line 70: Delete “being”.

Line 82: This should be written in the past not present tense.

Line 111 – 112: I would argue that this is unnecessary and should be deleted.

Line 115: I do not think that originates is the appropriate word here. The population does not breed in Amvarikos, it just forages here.

Line 128: “All samples were collected in a non-invasive way for the animal.” I would delete this sentence as you explain this in the methods.

Line 150: “In total, 25 samples were analyzed, twenty from the carapace and five from the skin.” This is a very confusing way to write this. I would change it to “In total, we collected 25 samples from loggerhead skin. In addition, for five of these animals we were also able to simultaneously sample their carapaces for diatoms.”

Table 1: This could be condensed as much information is repeated. How about one line for each sampling location with ranges?

Line 159-160: “Samples were processed following the methods described by Hasle and Syvertsen [30] for South African samples and van der Werff [31].” This is a confusing way to put this. Are you trying to say that you used the Hasle and Syvertsen method only for South African samples and the other method for all the other samples?

Line 172: Delete extra comma.

Line 190: “For pairwise comparisons of the loggerhead diatom flora from four localities, the Sørenson similarity index [36] was calculated.” should be “To make pair-wise comparison in between geographic location, we used the Sørenson similarity index”

Line 213: Include ‘diatom’ before taxa and delete ‘during the counts’.

Line 234: In my opinion, it is not appropriate in this context to refer to turtles as Green turtles or South African turtles. These turtles migrate between several different countries and so the term “sampled in Greece” is more accurate than “Greek turtle”.

Line 254-255: It is not clear what you are trying to say here.

Line 363: I think you mean exact richness of the population, or do you mean individual? It is not clear.

Line 366: “Will be” should be “would be”.

Line 364 – 367: This is a run-on sentence.

Line 373: I think you mean “predominantly” not “dominantly”.

Line 381: I would add ‘sampled so far’ to the end of this sentence as very few turtles have been sampled and so you cannot say this with confidence.

Line 380: Change “known” to “observed”.

Line 399: Run-on sentence.

Line 410: This makes it sound as if this was not found in non-Greek turtles, which were not sampled. Perhaps, rephrase to say “For Greek turtles, we sampled both skin and carapace. Interestingly, we found differences…”

Line 421: I am confused as to why you would expect to find obligate epibiont diatoms in the benthos. Surely, then they wouldn’t be obligate epibionts? Please explain.

Line 451: “As the biofilm accumulates, the available, uncolonized substratum surface on the carapace decreases and the relative abundance of strictly epizoic diatom taxa that needs this kind of substratum to thrive, declines” should be “As biofilm accumulates, the available and uncolonized substratum surface on the carapace decreases and so there will also be a decline in the relative abundance of strictly epizoic diatom taxa”.

Line 455: Two double spaces in the sentence.

Line 458: Double space in this sentence.

Line 460: Wandering is a very informal and inaccurate word in this context. “Satellite tracking of loggerheads showed that Amvrakikos Gulf turtles display long-term residency in this area, occasionaly wandering off to the northern Adriatic Sea using the latter as a foraging ground” should be “Satellite tracking have revealed that loggerhead turtles in Amvrakikos Gulf generally remain resident in this area but do occasionall venture to the northern Adriatic sea to forage”.

Line 490: Satellite tracking would be a more appropriate term than GPS tracking.
